# MYO9B gene polymorphisms are associated with the risk of inflammatory bowel diseases

**DOI:** 10.18632/oncotarget.11186

**Published:** 2016-08-10

**Authors:** Ming-Jie Wang, Xiao-Liang Xu, Guo-Liang Yao, Qiang Yu, Chun-Fu Zhu, Zhi-Jun Kong, Hui Zhao, Li-Ming Tang, Xi-Hu Qin

**Affiliations:** ^1^ Department of Orthopedics, Affiliated Hospital of Nanjing Medical University, Changzhou Second People's Hospital, Changzhou, China; ^2^ Liver Surgery of Jiangsu Province People's Hospital, The First Affiliated Hospital of Nanjing Medical University, Nanjing, China; ^3^ Department of Thoracic Surgery, The First Affiliated Hospital of Nanjing Medical University, Nanjing, China; ^4^ Department of General Surgery, Affiliated Hospital of Nanjing Medical University, Changzhou Second People's Hospital, Changzhou, China; ^5^ Department of General Surgery, Wuxi Third People's Hospital, Wuxi, China

**Keywords:** inflammatory bowel disease, Crohn's disease, ulcerative colitis, MYO9B, polymorphism, Immunology and Microbiology Section, Immune response, Immunity

## Abstract

*Myosin IXB* (*MYO9B*) gene polymorphisms have been extensively investigated in terms of their associations with inflammatory bowel disease (IBD), with contradictory results. The aim of this meta-analysis was to evaluate associations between *MY09B* gene polymorphisms and the risk of IBD, Crohn's disease (CD) and ulcerative colitis (UC). Eligible studies from PubMed, Embase, and CNKI databases were identified. Pooled odds ratios (ORs) and 95% confidence intervals (95% CIs) were calculated. Ten studies published in eight papers reporting 8,975 cases and 9,482 controls were included in this meta-analysis. Five *MY09B* gene polymorphisms were evaluated: rs1545620, rs962917, rs1457092, rs2305764, and rs2305767. Our data suggested that the rs1545620 polymorphism was associated with a decreased risk of IBD. A similar result was found for rs2305767 and UC. The rs962917 single nucleotide polymorphism (SNP) increased the risk of IBD, CD and UC. Moreover, rs1457092 increased the risk of IBD and UC. Rs2305764 was also associated with an increased risk of IBD. Furthermore, stratification analyses indicated that rs1545620 decreased the risk of IBD, while rs962917 increased the risk of IBD, CD and UC in Caucasian populations. To sum up, our data indicate that these five SNPs in *MY09B* are significantly associated with the risk of IBD.

## INTRODUCTION

Inflammatory bowel disease (IBD) is a chronic inflammatory disease of the intestinal tract that causes relapse and remission, and is usually classified into two clinical syndromes: ulcerative colitis (UC) and Crohn's disease (CD) [1]. Although the etiology of IBD is unclear, twin and familial aggregation studies have demonstrated roles for genetic factors. Genetic epidemiology studies have indicated that UC and CD are polygenic disorders, and that genetic susceptibility factors play an important role in the pathogenesis of these two diseases [2]. Previous studies identified several IBD susceptibility genes, including *nucleotide oligomerization domain 2 (NOD2)*, *interleukin-10 (IL-10)*, *IL-23*, and *ATG16L1* [3, 4]. Genome-wide association studies (GWAS) have implicated that genes were involved in both UC and CD, indicating a partly shared pathogenesis [5, 6].

*Myosin IXB* (*MYO9B*) encodes a single-headed processive myosin that is a member of the class IX myosin family [7]. MY09B is expressed in various tissues and cell types, including human intestinal epithelial cells [8]. Studies using a rat model shown that overexpression of MYO9B results in actin filament-related morphologic changes in epithelial cells [9]. A previous Dutch study first suggested *MYO9B* as a susceptibility gene for celiac disease [10]. *MYO9B* lies on chromosome 19p13.1, which has been linked to both celiac disease [11] and IBD [12]. Autoimmune-related disorders may possess some common genetic susceptibility factors [13]. Furthermore, *MYO9B* has been shown to be associated with IBD [12]. Therefore, *MYO9B* may be a candidate susceptibility gene for IBD.

Recently, numerous studies investigated associations between *MYO9B* gene polymorphisms and IBD susceptibility, but with discordant results [14-21]. The clinical heterogeneity, different ethnic populations and small sample sizes of previous studies may have contributed to these disparities. To overcome these limitations and resolve inconsistencies, we performed a meta-analysis of the contradictory results from these relevant studies to clarify the associations between *MY09B* gene polymorphisms and risk of IBD, CD and UC.

## RESULTS

### Characteristics of the included studies

**Figure 1:**
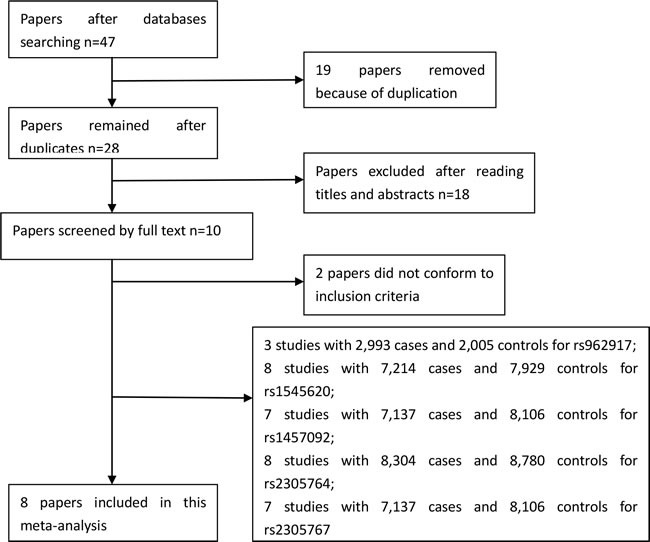
Selection for eligible studies included in this meta-analysis

A total of 47 papers were identified after our initial search. After removing duplicates and screening the titles and abstracts, 37 papers were removed. Therefore, 10 papers were selected for further full text review; two papers [22, 23] were excluded, because they did not investigate the associations between *MY09B* gene polymorphisms and IBD, CD or UC. We finally identified eight eligible papers [14-21] including ten studies (8,975 cases and 9,482 controls) in this meta-analysis. Selection for included studies was presented in Figure 1. The characteristics of these included studies are summarized in Table 1. These studies were published from 2006 to 2014. The number for each genotype about five SNPs of *MY09B* was showed Supplementary Table 1. Five SNPs within *MY09B* gene were investigated, including rs1545620, rs962917, rs1457092, rs2305764 and rs2305767. Genotype distributions of the controls about rs1545620 in one study [17] did not conform to HWE [17] (*P* = 0.002). The NOS scores of all included studies ranged from 5 to 7 stars, which suggested they were studies of high methodological quality. Six papers were carried out in Caucasian populations [14-16, 18, 20, 21], and two in Asian populations [17, 19].

**Table 1:** Characteristics of included studies

Author and year	Country	Ethnicity	Numbers			Polymorphisms	HWE values	Genotype methods	NOS score
			UC	CD	Controls				
Hu_2014	China	Asian	235	207	402	rs962917,rs1545620	0.252, 0.002	PCR	7
Shi_2011	China	Asian	245	NA	300	rs1545620	0.310	PCR	6
Wolters_2011	Canada	Caucasian	603	754	924	rs962917,rs1457092,rs1545620,rs2305764,rs2305767	0.971, 0.971, 0.992, 0.992, 0.995	TaqMan	6
Cooney_2009	UK	Caucasian	650	652	1190	rs1457092,rs1545620,rs2305764,rs2305767	0.988, 0.977, 0.987, 0.993	MALDI-TOF	6
Latiano_2008	Italy	Caucasian	658	549	674	rs962917,rs1545620,rs2305764	0.101, 0.114, 0.107	PCR	5
Nunez_2007	Spain	Caucasian	677	627	990	rs1457092,rs2305764,rs2305767	0.584, 0.772, 0.051	NA	7
van Bodegraven_2006	Dutch	Caucasian	290	298	1624	rs1457092,rs1545620,rs2305764,rs2305767	0.992, 0.992, 0.995, 0.991	TaqMan	6
van Bodegraven_2006	UK	Caucasian	580	735	2371	rs1457092,rs1545620,rs2305764,rs2305767	0.994, 0.997, 0.986, 0.980	TaqMan	6
van Bodegraven_2006	Canada/Italy	Caucasian	650	164	445	rs1457092,rs1545620,rs2305764,rs2305767	0.969, 0.960,0.994, 1.000	TaqMan	6
Amundsen_2006	Norway	Caucasian	308	149	562	rs1457092,rs2305764,rs2305767	0.974, 0.972, 0.960	NA	6

Abbreviations: CD, Crohn's disease; UC, ulcerative disease; NA, not available; NOS, Newcastle–Ottawa scale.

### Meta-analysis of rs1545620

**Figure 2:**
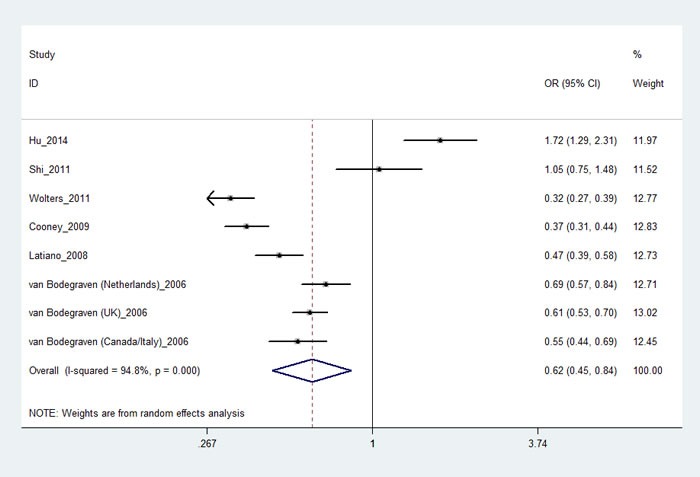
Forest plot shows odds ratio for associations between the rs1545620 polymorphism and IBD (CC *vs.* AA+AC)

Six papers [15-19, 21] including eight studies with 7,214 cases and 7,929 controls examined rs1545620 polymorphism. As shown in Table 2, rs1545620 polymorphism was associated with a decreased risk of IBD in recessive model (CC *vs*. AA+AC: OR, 0.62; 95% CI, 0.45-0.84, *P* = 0.002, Figure 2); however, our study did not find rs1545620 was associated with UC or CD risk. Stratification analyses were conducted according to ethnicity. The results indicated that rs1545620 polymorphism was also significantly associated with a decreased risk of IBD among Caucasian populations in recessive model (CC *vs*. AA+AC: OR, 0.48; 95% CI, 0.38-0.62, *P* < 0.001). We did not find rs1545620 was related with UC or CD risk in Caucasian or Asian populations. By exclusion of one Chinese study that did not conform to HWE [17], the pooled estimates of the remaining studies also showed that rs1545620 polymorphism might decrease the risk of IBD (CC *vs*. AA+AC: OR, 0.53; 95% CI, 0.41-0.69, *P* < 0.001) (Table 3), suggesting that the results of this SNP was stable. The power analysis showed that our study has a power of 99% to detect the effects of rs1545620 polymorphism on IBD susceptibility, assuming an OR of 0.62. The false positive report probability (FPRP) of four genetic models were less than 0.5, indicating our data about this polymorphism was trustworthy.

**Table 2:** Meta-analysis of associations between the rs1545620 polymorphism and IBD, UC, CD

Disease	Comparison	Populations	Studies	OR (95% CI)	*P*-value	*P* for heterogeneity	Model	*I*^2^ (%)	P_Begger_P_Egger_ for Publication bias
IBD	C vs. A	Overall	8	0.95(0.86,1.06)	0.391	<0.001	R	78.4	0.322/0.568
		Caucasian	6	0.94(0.83,1.06)	0.301	<0.001	R	83.2	Na/Na
		Asian	2	1.03 (0.81,1.32)	0.793	0.167	F	47.6	Na/Na
	CC vs. AA+AC	Overall	8	0.62(0.45,0.84)	0.002	<0.001	R	94.8	0.083/0.169
		Caucasian	6	0.48(0.38,0.62)	<0.001	<0.001	R	90.4	Na/Na
		Asian	2	1.36(0.84,2.20)	0.212	0.032	R	78.1	Na/Na
	CC+AC vs. AA	Overall	8	0.91(0.78,1.08)	0.287	0.001	R	72.0	0.805/0.723
		Caucasian	6	0.91(0.78,1.05)	0.199	0.007	R	68.8	Na/Na
		Asian	2	1.49(0.30,7.48)	0.625	0.003	R	88.9	Na/Na
	CC vs. AA	Overall	8	0.92(0.72,1.18)	0.527	<0.001	R	81.7	0.458/0.412
		Caucasian	6	0.88(0.68,1.14)	0.348	<0.001	R	83.5	Na/Na
		Asian	2	1.51(0.33,6.90)	0.596	0.005	R	87.3	Na/Na
CD	C vs. A	Overall	7	0.95(0.85,1.06)	0.335	0.006	R	67.1	0.881/0.709
		Caucasian	6	0.94(0.84,1.07)	0.362	0.003	R	72.5	Na/Na
		Asian	1	0.97(0.75,1.27)	0.842	Na	Na	Na	Na/Na
	CC vs. AA+AC	Overall	7	0.96(0.81,1.13)	0.607	0.010	R	64.3	0.652/0.373
		Caucasian	6	0.95(0.78,1.15)	0.572	0.006	R	69.4	Na/Na
		Asian	1	1.04(0.74,1.46)	0.807	Na	Na	Na	Na/Na
	CC+AC vs. AA	Overall	7	0.91(0.83,1.01)	0.069	0.145	F	37.2	0.881/0.754
		Caucasian	6	0.91(0.79,1.05)	0.217	0.096	R	46.6	Na/Na
		Asian	1	0.81(0.48,1.37)	0.434	Na	Na	Na	Na/Na
	CC vs. AA	Overall	7	0.89(0.71,1.13)	0.346	0.004	R	68.5	0.881/0.827
		Caucasian	6	0.90(0.70,1.17)	0.425	0.002	R	73.3	Na/Na
		Asian	1	0.85(0.49,1.45)	0.545	Na	Na	Na	Na/Na
UC	C vs. A	Overall	8	0.91(0.81,1.02)	0.108	0.001	R	71.4	0.805/0.758
		Caucasian	6	0.92(0.80,1.06)	0.237	<0.001	R	79.2	Na/Na
		Asian	2	0.87(0.72,1.05)	0.141	0.749	F	0.0	Na/Na
	CC vs. AA+AC	Overall	8	0.89(0.73,1.08)	0.247	0.001	R	73.0	0.216/0.642
		Caucasian	6	0.91(0.74,1.12)	0.378	0.001	R	74.8	Na/Na
		Asian	2	0.58(0.16,2.08)	0.405	0.013	R	83.6	Na/Na
	CC+AC vs. AA	Overall	8	0.88(0.76,1.01)	0.073	0.029	R	55.2	0.621/0.253
		Caucasian	6	0.89(0.75,1.05)	0.163	0.021	R	62.3	Na/Na
		Asian	2	0.80(0.53,1.19)	0.270	0.161	F	49.1	Na/Na
	CC vs. AA	Overall	8	0.79(0.60,1.03)	0.078	<0.001	R	76.7	0.621/0.340
		Caucasian	6	0.85(0.64,1.14)	0.278	<0.001	R	79.8	Na/Na
		Asian	2	0.49(0.22,1.10)	0.085	0.130	F	56.3	Na/Na

* Bold values are statistically significant (P < 0.05). Abbreviations: OR, odds ratio; 95% CI, 95% confidence interval; CD, Crohn's disease; UC, ulcerative disease; IBD, inflammatory bowel disease; F, fixed effects model; R, random effects model; Na, not available.

**Table 3:** Meta-analysis of the association between the rs1545620 polymorphism and IBD, UC, CD susceptibility after excluding one study (Hu et al.)

Disease	Comparison	Model	OR(95%CI)	*P*	*I*^2^ (%)	P for heterogeneity
IBD	C vs. A	R	0.96(0.85,1.08)	0.473	81.5	<0.001
	CC vs. AA+AC	R	0.53(0.41,0.69)	<0.001	91.5	<0.001
	CC+AC vs. AA	R	0.94(0.79,1.12)	0.470	74.2	0.001
	CC vs. AA	R	0.95(0.72,1.25)	0.720	84.1	<0.001
CD	C vs. A	R	0.94(0.84,1.07)	0.362	72.5	0.003
	CC vs. AA+AC	R	0.95(0.78,1.15)	0.572	69.4	0.006
	CC+AC vs. AA	R	0.91(0.79,1.05)	0.217	46.6	0.096
	CC vs. AA	R	0.90(0.70,1.17)	0.425	73.7	0.002
UC	C vs. A	R	0.91(0.81,1.03)	0.152	75.4	<0.001
	CC vs. AA+AC	R	0.87(0.70,1.08)	0.218	76.1	<0.001
	CC+AC vs. AA	R	0.90(0.78,1.04)	0.147	55.0	0.038
	CC vs. AA	R	0.80(0.60,1.07)	0.136	79.5	<0.001

* Bold values are statistically significant (*P* < 0.05). Abbreviations: OR, odds ratio; 95% CI, 95% confidence interval; CD, Crohn's disease; UC, ulcerative disease; IBD, inflammatory bowel disease; F, fixed effects model; R, random effects model.

### Meta-analysis of rs962917

**Figure 3:**
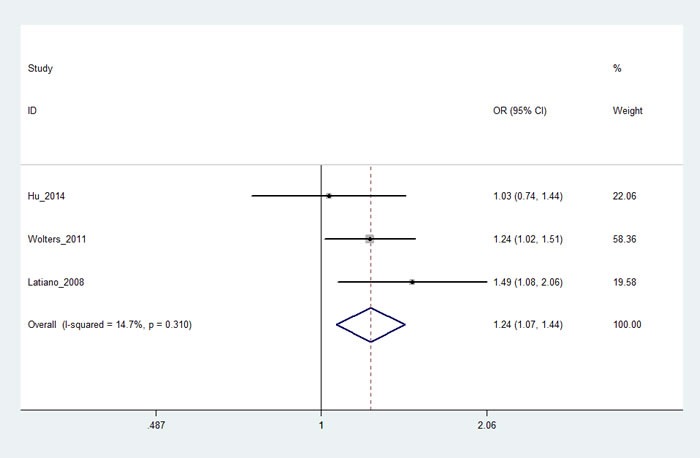
Forest plot shows odds ratio for the associations between rs962917 and CD(AA *vs.* GG+GA)

Three studies [16, 17, 21] with 2,993 cases and 2,005 controls investigated rs962917 polymorphism. As shown in Table 4, rs962917 polymorphism was associated with an increased risk of IBD. Stratification analyses of ethnicity also indicated that rs962917 polymorphism was significantly associated with the increased risk of IBD among Caucasian populations. We also found rs962917 was related with UC or CD risk (AA *vs*. GG+GA: OR, 1.24; 95% CI, 1.07-1.44, *P* = 0.005, Figure 3), especially among Caucasian populations, but not Asian populations. We did not perform sensitivity analyses, because only three studies investigated rs962917 polymorphism. Due to limited studies, we did not conduct Egger's and Begg's tests in stratification analyses of ethnicity. Both Egger's and Begg's tests revealed that there was no obvious publication bias in overall analysis for rs962917 polymorphism. The power of this meta-analysis about rs962917 polymorphism ranged from 0.57 to 0.97.

The FPRP of four genetic models was less than 0.5, indicating our data about this polymorphism was trustworthy.

**Table 4:** Meta-analysis of associations between the rs962917 polymorphism and IBD, UC, CD

Disease	Comparison	Populations	Studies	OR (95% CI)	*P*-value	*P* for heterogeneity	Model	*I*^2^ (%)	P_Begger_/P_Egger_ for Publication bias
IBD	A vs. G	Overall	3	1.13(1.04,1.23)	0.003	0.667	F	0.0	0.602/0.400
		Caucasian	2	1.15(1.05,1.26)	0.002	0.727	F	0.0	Na/Na
		Asian	1	1.04(0.84,1.29)	0.695	Na	Na	Na	Na/Na
	AA vs. GG+GA	Overall	3	1.26(1.10,1.43)	<0.001	0.130	F	51.1	0.117/0.595
		Caucasian	2	1.28(1.11,1.48)	0.001	0.055	R	72.9	Na/Na
		Asian	1	1.16(0.88,1.52)	0.285	Na	Na	Na	Na/Na
	AA+GA vs. GG	Overall	3	1.08(0.94,1.25)	0.281	0.273	F	23.0	0.602/0.460
		Caucasian	2	1.12(0.96,1.30)	0.148	0.467	F	0.0	Na/Na
		Asian	1	0.76(0.45,1.26)	0.284	Na	Na	Na	Na/Na
	AA vs. GG	Overall	3	1.31(1.10,1.58)	0.003	0.118	F	53.2	0.602/0.445
		Caucasian	2	1.40(1.15,1.70)	0.001	0.335	F	53.2	Na/Na
		Asian	1	0.83(0.49,1.40)	0.481	Na	Na	Na	Na/Na
UC	A vs. G	Overall	3	1.12(1.01,1.24)	0.025	0.426	F	0.0	0.602/0.830
		Caucasian	2	1.12(1.00,1.24)	0.048	0.199	F	39.3	Na/Na
		Asian	1	1.15(0.89,1.49)	0.279	Na	Na	Na	Na/Na
	AA vs. GG+GA	Overall	3	1.30(0.97,1.74)	0.075	0.037	R	69.7	0.602/0.415
		Caucasian	2	1.33(0.83,2.13)	0.243	0.010	R	84.8	Na/Na
		Asian	1	1.28(0.93,1.77)	0.132	Na	Na	Na	Na/Na
	AA+GA vs. GG	Overall	3	1.06(0.89,1.25)	0.508	0.884	F	0.0	0.117/0.068
		Caucasian	2	1.07(0.89,1.25)	0.443	0.921	F	0.0	Na/Na
		Asian	1	0.91(0.49,1.71)	0.772	Na	Na	Na	Na/Na
	AA vs. GG	Overall	3	1.29(1.04,1.30)	0.020	0.151	F	47.1	0.602/0.800
		Caucasian	2	0.98(0.83,1.15)	0.169	0.071	R	69.2	Na/Na
		Asian	1	0.83(0.66,1.04)	0.135	Na	Na	Na	Na/Na
CD	A vs. G	Overall	3	1.12(1.00,1.28)	0.044	0.289	F	19.3	0.117/0. 107
		Caucasian	2	1.17(1.05,1.30)	0.005	0.703	F	0.0	Na/Na
		Asian	1	0.94(0.72,1.22)	0.622	Na	Na	Na	Na/Na
	AA vs. GG+GA	Overall	3	1.24(1.07,1.44)	0.005	0.310	F	14.7	0.602/0.989
		Caucasian	2	1.30(1.10,1.54)	0.002	0.339	F	0.0	Na/Na
		Asian	1	1.03(0.74,1.44)	0.846	Na	Na	Na	Na/Na
	AA+GA vs. GG	Overall	3	1.04(0.78,1.40)	0.780	0.099	R	56.8	0.602/0.476
		Caucasian	2	1.14(0.96,1.37)	0.142	0.294	F	9.1	Na/Na
		Asian	1	0.63(0.35,1.14)	0.128	Na	Na	Na	Na/Na
	AA vs. GG	Overall	3	1.21(0.83,1.76)	0.323	0.074	R	61.7	0.602/0.179
		Caucasian	2	1.43(1.14,1.80)	0.002	0.901	F	0.0	Na/Na
		Asian	1	0.83(0.66,1.04)	0.194	Na	Na	Na	Na/Na

* Bold values are statistically significant (***P*** < 0.05). Abbreviations: OR, odds ratio; 95% CI, 95% confidence interval; CD, Crohn's disease; UC, ulcerative disease; IBD, inflammatory bowel disease; F, fixed effects model; R, random effects model; Na, not available.

### Meta-analysis of rs1457092, rs2305764, rs2305767

**Figure 4:**
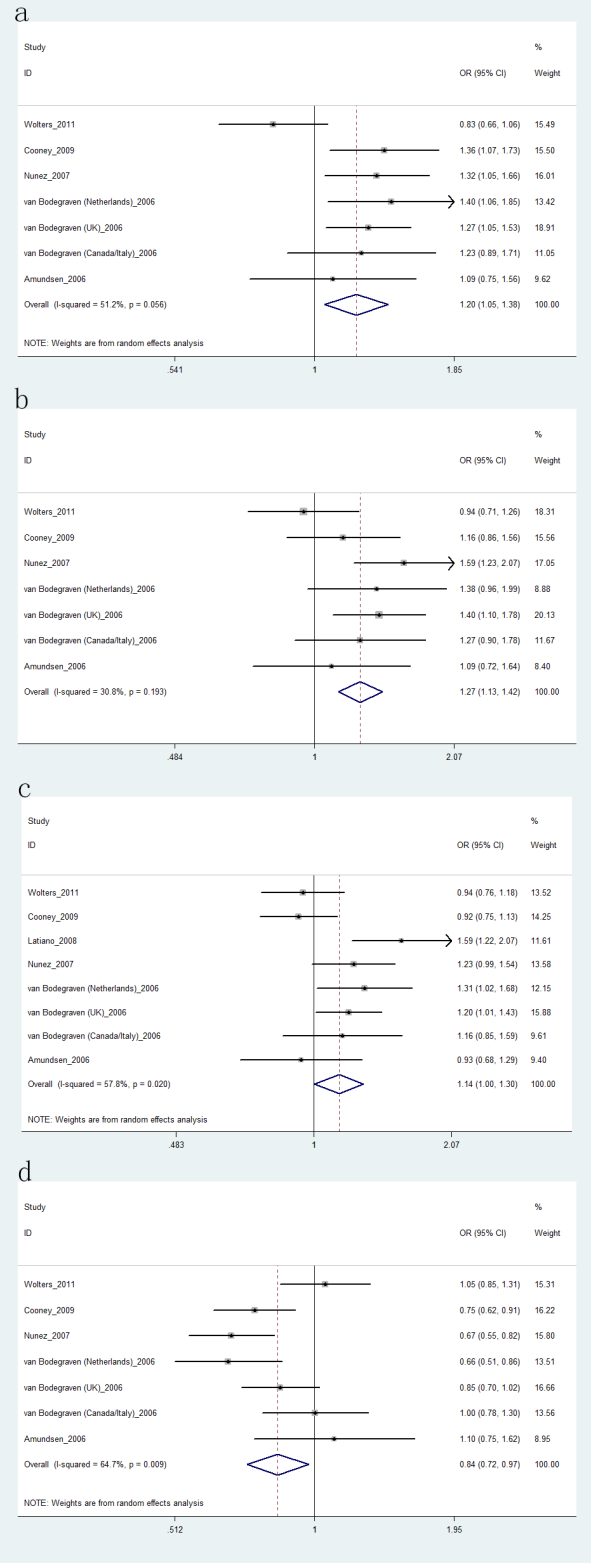
Forest plot shows odds ratio for the associations **a.** between rs1457092 polymorphism and risk of IBD (AA ***vs.*** CC+CA); **b.** between rs1457092 polymorphism and risk of UC (AA *vs*. CC+CA); **c.** between the rs2305764 polymorphism and risk of IBD (AA *vs*. GG+GA); **d.** between rs2305767 polymorphism and risk of UC (GG+AG *vs*. AA).

Five papers [14, 15, 18, 20, 21] including seven studies with 7,137 cases and 8,106 controls examined rs1457092 polymorphism; six papers [14-16, 18, 20, 21] including eight studies with 8,304 cases and 8,780 controls studied rs2305764 polymorphism; five papers [14, 15, 18, 20, 21] including seven studies with 7,137 cases and 8,106 controls studied rs2305767 polymorphism. For these three SNPs, we did not perform stratification analyses due to lack of data. As shown in Table 5, we found rs1457092 increased the risk of IBD (AA *vs*. CC+CA: OR, 1.20; 95% CI, 1.05-1.38, *P* = 0.009, Figure 4a) or UC (Figure 4b), but not CD. For rs2305764, we also found rs2305764 increased the risk of IBD in recessive model (AA *vs*. GG+GA: OR, 1.14; 95% CI, 1.00-1.30, *P* = 0.045, Figure 4c). With regard to rs2305767, our results suggested rs2305767 was associated with a decreased risk of UC (GG+AG *vs*. AA: OR, 0.84; 95% CI, 0.72-0.97, *P* = 0.020, Figure 4d), but not associated with IBD and CD risk. The power analysis showed that the power of this meta-analysis with regard to rs1457092, rs2305764, rs2305767 polymorphisms was greater than 0.95. The FPRP of genetic models were less than 0.5, indicating our data about these polymorphisms was trustworthy.

**Table 5:** Meta-analysis of associations between rs1457092, rs2305764, rs2305767 polymorphisms and IBD, UC, CD

Polymorphism	Comparison	Disease	Studies	OR (95% CI)	*P*-value	*P* for heterogeneity	Model	*I*^2^ (%)	P_Begger_P_Egger_ for Publication bias
rs1457092	A vs. C	IBD	7	1.11(1.01,1.21)	0.029	0.001	R	73.1	0.652/0.837
		UC	7	1.15(1.08,1.22)	<0.001	0.120	F	40.7	0.293/0.711
		CD	7	1.05(0.93,1.19)	0.402	0.001	R	73.3	0.652/0.977
	AA vs. CC+CA	IBD	7	1.20(1.05,1.38)	0.009	0.056	R	51.2	0.652/0.822
		UC	7	1.27(1.13,1.42)	<0.001	0.193	F	30.8	0.453/0.413
		CD	7	0.92(0.54,1.55)	0.745	<0.001	R	94.6	0.099/0.104
	AA+CA vs. CC	IBD	7	1.16(1.06,1.26)	0.001	0.287	F	18.7	0.453/0.868
		UC	7	1.16(1.06,1.28)	0.001	0.204	F	30.9	0.453/0.989
		CD	7	1.05(0.90,1.24)	0.528	0.003	R	69.8	0.881/0.918
	AA vs. CC	IBD	7	1.25(1.04,1.51)	0.018	0.004	R	68.2	0.652/0.851
		UC	7	1.34(1.18,1.51)	<0.001	0.128	F	39.6	0.176/0.532
		CD	7	1.13(0.89,1.42)	0.316	0.007	R	66.4	0.881/0.984
rs2305764	A vs. G	IBD	8	1.06(0.99,1.14)	0.084	0.026	R	56.1	1.000/0.918
		UC	8	0.97(0.71,1.34)	0.867	<0.001	R	96.9	0.138/0.688
		CD	8	0.71(0.45,1.13)	0.150	<0.001	R	98.3	0.322/0.166
	AA vs. GG+GA	IBD	8	1.14(1.00,1.30)	0.045	0.020	R	57.8	0.621/0.861
		UC	8	1.01(0.69,1.47)	0.966	<0.001	R	92.1	0.048/0.054
		CD	8	0.94(0.67,1.33)	0.739	<0.001	R	86.6	0.621/0.084
	AA+GA vs. GG	IBD	8	1.05(0.98,1.12)	0.145	0.134	F	37.0	0.458/0.853
		UC	8	0.95(0.63,1.43)	0.800	<0.001	R	96.2	0.805/0.495
		CD	8	0.66(0.35,1.26)	0.209	<0.001	R	98.2	0.322/0.407
	AA vs. GG	IBD	8	1.15(0.99,1.34)	0.061	0.015	R	59.6	0.621/0.993
		UC	8	0.98(0.58,1.63)	0.928	<0.001	R	94.8	0.048/0.080
		CD	8	0.83(0.53,1.30)	0.413	<0.001	R	90.9	0.621/0.068
rs2305767	G vs. A	IBD	7	0.92(0.83,1.02)	0.113	<0.001	R	79.1	0.652/0.964
		UC	7	0.89(0.80,0.99)	0.032	0.005	R	67.8	0.652/0.577
		CD	7	0.98(0.89,1.07)	0.622	0.024	R	58.9	0.652/0.379
	GG vs. AA+AG	IBD	7	0.91(0.78,1.06)	0.240	0.004	R	68.5	0.453/0.644
		UC	7	0.89(0.77,1.03)	0.124	0.089	R	45.3	0.881/0.468
		CD	7	1.00(0.90,1.11)	0.972	0.145	F	37.1	0.293/0.259
	GG+AG vs. AA	IBD	7	0.88(0.77,1.01)	0.067	0.002	R	71.9	0.881/0.837
		UC	7	0.84(0.72,0.97)	0.020	0.009	R	64.7	0.881/0.441
		CD	7	0.98(0.89,1.07)	0.601	0.119	F	40.7	0.881/0.500
	GG vs. AA	IBD	7	0.85(0.69,1.05)	0.134	<0.001	R	77.6	0.453/0.937
		UC	7	0.81(0.66,0.99)	0.039	0.014	R	62.4	0.881/0.743
		CD	7	0.96(0.79,1.17)	0.675	0.029	R	57.2	0.453/0.351

* Bold values are statistically significant (P < 0.05). Abbreviations: OR, odds ratio; 95% CI, 95% confidence interval; CD, Crohn's disease; UC, ulcerative disease; IBD, inflammatory bowel disease; F, fixed effects model; R, random effects model.

### Sensitivity analysis and publication bias

**Figure 5:**
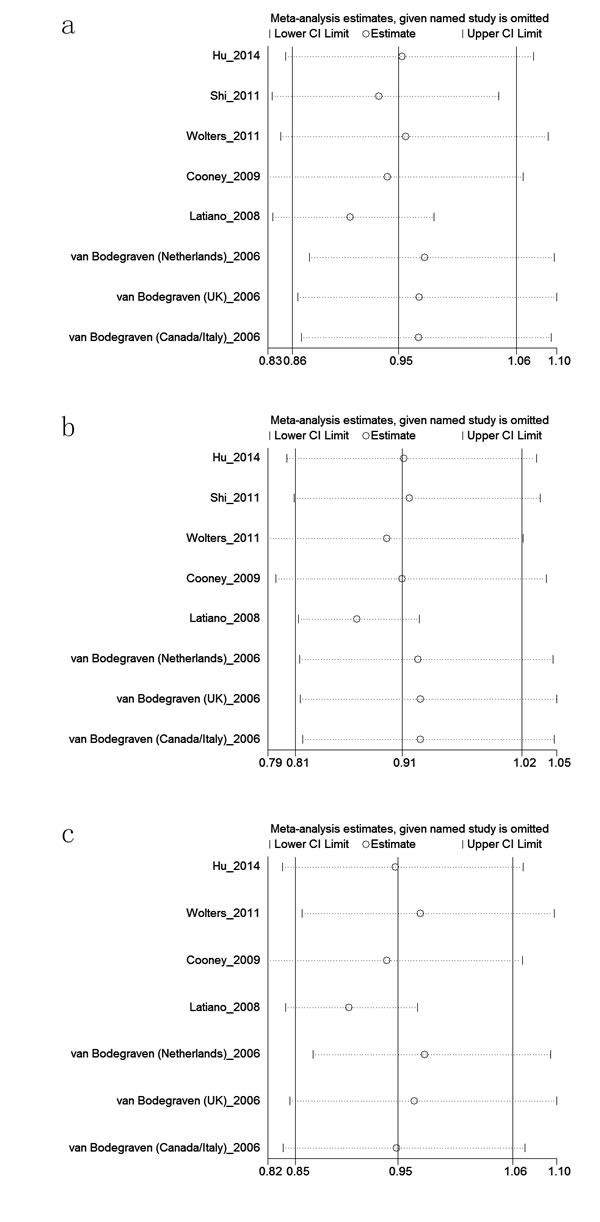
Sensitivity analyses between the rs1545620 polymorphism and (a): IBD, (b): UC and (c): CD risk in allele model

We assessed sensitivity by omitting each study once at a time in every genetic model for these five SNPs. The pooled ORs for the effects of rs962917, rs1457092, rs2305764 and rs2305767 on the risk for IBD or CD or UC indicated that our data were stable and trustworthy about these four SNPs. However, we found pooled ORs for the effect of rs1545620 on the risk for IBD (Figure 5a) or CD (Figure 5b) or UC (Figure 5c) changed significantly when we omitted the study [16] by Latiano et al., suggesting that our data about rs1545620 polymorphism were poorly stable and trustworthy.

**Figure 6:**
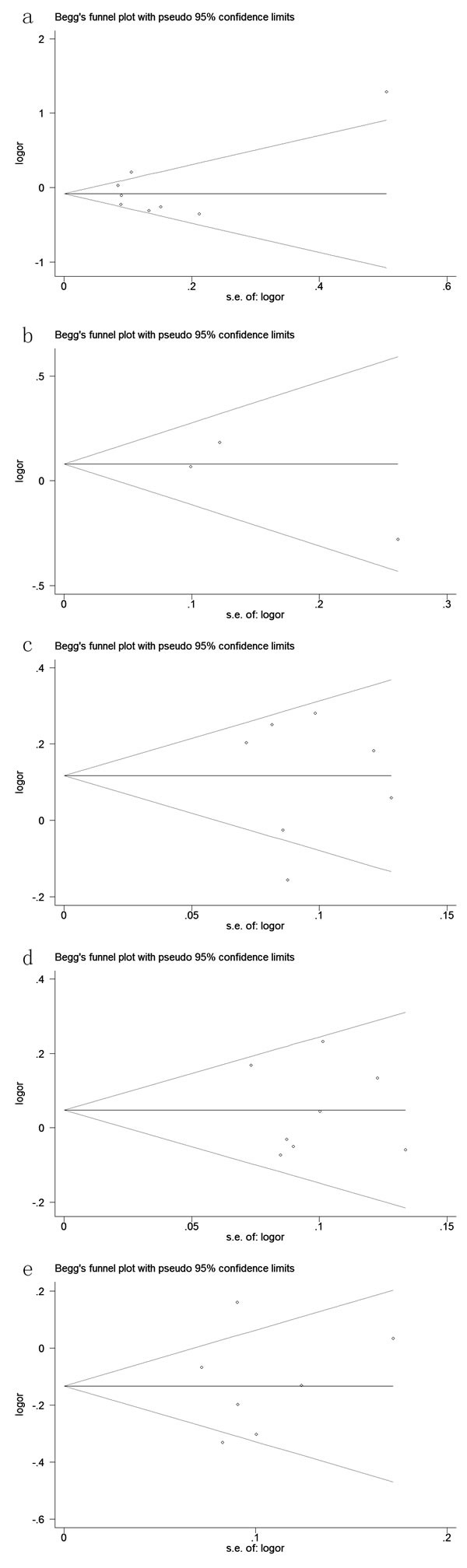
Begg's tests for publication bias between (a): rs1545620, (b): rs962917, (c): rs1457092, (d): rs2305764 and (e): rs2305767 polymorphisms and the risk of IBD in dominant model

Both Egger's and Begg's tests were used to evaluated the publication bias of this meta-analysis. Our data revealed that there was no obvious publication bias in overall analysis for these five SNPs (Table 2, 4, 5 and Figure 6). Due to limited studies, we did not conduct Egger's and Begg's tests in stratification analyses of ethnicity.

## DISCUSSION

In this meta-analysis, we evaluated recent studies on the relationships between *MY09B* gene polymorphisms and susceptibility to IBD, CD and UC. The primary findings were as follows: (1) the rs1545620 polymorphism was associated with a decreased risk of IBD; (2)rs2305767 decreased the risk of UC; (3) rs962917 increased the risk of IBD, CD and UC; (4) rs1457092 increased the risk of IBD and UC; (5) rs2305764 was associated with an increased risk of IBD; and (6) stratification analyses indicated that rs1545620 decreased the risk of IBD in Caucasian populations, while rs962917 increased the risk of IBD, CD and UC in Caucasian populations.

*MYO9B* is a candidate gene that is reportedly associated with celiac disease [10] and diabetes type 1 (DM type 1) [24, 25]. A previous study [26] demonstrated increased intestinal permeability in DM type 1 patients *in vivo*, which was associated with increased *MYO9B* gene expression. Their findings indicated a close link between *MYO9B* expression and changes in intestinal permeability [26]. MYO9B is a single motor protein with a Rho GTPase-activating domain, which downregulates Rho-dependent signaling pathways by converting active Rho-GTP into inactive Rho-GDP [9]. GTPases of the Rho family have been demonstrated to be involved in the regulation of tight junction function and actin filament remodeling, which lead to enhanced paracellular epithelial permeability [27, 28]. Therefore, it is reasonable to hypothesize that MYO9B may cause impaired intestinal barrier integrity. Furthermore, IBD is often characterized by increased permeability of intestinal epithelium [29, 30]. These findings suggest that MY09B may play an important role in the pathogenesis of IBD, and that *MY09B* is a candidate susceptibility gene for IBD.

To date, several studies have assessed associations between *MYO9B* gene polymorphisms and IBD susceptibility; however, they reported conflicting results. Several studies [14, 17, 19, 21] did not find *MYO9B* to be a candidate gene for IBD, while other studies [15, 16, 18, 20] suggested this gene was significantly associated with IBD, CD or UC risk. However, these studies had inadequate statistical power and involved relatively small samples. To overcome these limitations, we conducted a meta-analysis to assess associations between *MY09B* gene polymorphisms and the risk of IBD, CD and UC.

We determined that rs1545620 was associated with a decreased risk of IBD and rs2305767 was associated with a decreased risk of UC. Moreover, three other SNPs (rs962917, rs1457092 and rs2305764) increased the risk of IBD, CD or UC. It is noteworthy that only rs962917 was related to the risk of CD in this meta-analysis, while the four other SNPs were not. We found that rs1457092 and rs2305767 were associated with the risk of UC. Previous studies have demonstrated genetic differences between CD and UC [31]. Twin concordance rate data have indicated that the heritable component is less crucial for UC than for CD [32].A Spanish study [20] by Nunez et al. found that *MYO9B* gene polymorphisms were not correlated with CD, but were with UC. Van Bodegraven et al. reported that *MY09B* was more strongly associated with UC than with CD [18]. Furthermore, they found *MY09B* gene polymorphisms were not associated with CD in the Canadian/Italian populations [18]. It is therefore reasonable to assume that *MYO9B* may affect susceptibility to UC specifically. With regard to rs962917, only three studies [16, 17, 21] involving 1,500 cases and 2,005 controls addressed the association between this SNP and CD; however, these studies involved a smaller sample size compared with works focusing on the other four SNPs. Any association between SNPs in genes with diseases is greatly affected by the number of subjects. Given the small number of participants, we should interpret the relationship between rs962917 and CD with caution. Furthermore, the power of this meta-analysis about rs962917 polymorphism was smaller compared with other four polymorphisms. The power of rs1457092, rs2305764, rs2305767 and rs1545620 polymorphisms was greater than 0.95, which suggested that our data were trustworthy. Further studies on the role of *MY09B* in UC are warranted.

Analyses stratified by ethnicity indicated that rs1545620 decreased the risk of IBD in Caucasian populations. Our data also suggest that rs962917 increased the risk of IBD, CD and UC in Caucasian populations. However, a relationship between *MY09B* and IBD, CD or UC was not detected in Asian populations. The genetic background of IBD may vary among ethnicities. Previous studies have demonstrated *OCTN* or *CARD15* gene variations to be associated with susceptibility to CD in Caucasian populations [33, 34], but not in Asian populations [35]. Two Asian studies from China [17, 19] reported no associations between *MY09B* gene polymorphisms and IBD. Although Hu et al. revealed that *MYO9B* gene might influence the sub-phenotypic expression of CD, they did not find an association between these *MYO9B* polymorphisms and intestinal permeability in IBD [17]. It is intriguing to hypothesize that variations in *MYO9B* predispose Caucasians to IBD. However, we cannot definitively state that *MYO9B* is not related to IBD in Asian patients, because this meta-analysis included only two Chinese studies [17, 19] with limited sample sizes. Larger-scale studies are urgently needed to assess associations between *MYO9B* gene polymorphisms and IBD in Asian populations and other ethnicities.

Among all included studies, one Chinese study [17] did not conform to HWE with respect to the rs1545620 polymorphism. After excluding this study, the pooled estimates of the remaining studies indicated that the rs1545620 polymorphism might decrease the risk of IBD (Table 3), consistent with the initial result. Heterogeneity was not reduced after exclusion of the Chinese study; it is thus tempting to speculate that this may not have been a source of heterogeneity. A sensitivity analysis of rs1545620 omitting the study by Latiano et al. [16] significantly altered the pooled ORs for the effect of this SNP on the risk of IBD, CD or UC, suggesting that the rs1545620 data are poorly reliable. It is noting that this study [16] did not provide the basic data of controls. We did not make sure whether the controls in this study were matched cases for age, sex or other confounders. As a result, we could not exclude the possibility that their results were false positive. The reasons why this study [16] affected the results of rs1545620 polymorphism remained unclear, but may be partially explained by above assumption. As for other reasons, clinical heterogeneity, and different ethnic populations may explain it, which needs further verification.

Several potential limitations of this meta-analysis should be taken into consideration. First, one Chinese study did not conform to HWE with respect to rs1545620 polymorphism; however, our data indicated the results of this SNP were trustworthy. Second, the numbers of studies included in the meta-analysis was small, and the sample size of this meta-analysis was not large enough. Third, our results were based on unadjusted estimates, without considering other confounders (such as age, gender, or environmental factors); thus, a more precise analysis should be performed, assuming the availability of individual data. Fourth, due to inclusion of only two Asian studies, we could not conduct analyses on rs1457092, rs2305764 or rs2305767 stratified by ethnicity. Future studies that include Asian populations and other ethnic groups are warranted due to ethnic differences in gene polymorphisms. Fifth, the heterogeneity of this meta-analysis was high in some genetic models.

In conclusion, this meta-analysis indicated that five SNPs within *MY09B* were associated with the risk of IBD, CD or UC. Future large-scale studies in Asian populations and other ethnicities are urgently needed to more accurately characterize associations between *MY09B* gene polymorphisms and IBD, CD and UC.

## MATERIALS AND METHODS

### Literature search

We performed a comprehensive search in PubMed, Embase, and CNKI databases to identify studies through January 1, 2016 that were related with the *MY09B* gene polymorphisms and IBD, CD or UC. The following search terms were used: “inflammatory bowel disease,” “IBD” “ulcerative disease,” “UC,” “Crohn's disease,” “CD,” “MYO9B,” “myosin IXB” and “polymorphisms”. Two independent authors conducted the search. No language or other restrictions were placed on the search. We also performed a manual search of references cited in published articles to identify other initially omitted studies. Any disagreements were resolved by consensus.

### Criteria of inclusion and exclusion

Criteria for the inclusion in this analysis were: (1) studies that evaluated the associations between UC or CD or IBD with at least one of the five single nucleotide polymorphisms (SNPs), (2) studied on human beings, (3) study provided sufficient data to calculate the Odds ratios (ORs) and 95% confidence intervals (CIs), and *P* value, and (4) case-control study. Exclusion criteria were: (1) duplication of previous publications; (2) review, editorial, or other non-original studies; (3) studies without detailed genotype data; (4) studies were without control group.

### Data extraction and quality assessment

For all eligible studies, the extracted information including: name of first author, publication year, country of origin, ethnicity, numbers of cases (UC and CD) and controls, and polymorphisms. Two authors independently performed the extraction of data and evaluated the study quality based on the Newcastle-Ottawa Scale (NOS) [36]. Total NOS scores ranged from 0 to 9. A score ranging 5 to 9 stars is considered to be a generally high methodological quality whereas a score ranging 0 to 4 is regarded as a relatively poor quality [37]. They compared results and agreed on a consensus; disagreements were resolved by discussion.

### Statistical analysis

All statistical analyses were performed using the Stata 11.0 software (StataCorp, College Station, TX, USA). Pooled ORs and 95% CIs were calculated to assess the associations between UC or CD or IBD and the five polymorphisms of *MY09B* gene. *P* < 0.05 was considered statistically significant. Heterogeneity was evaluated by the Q statistic (significant at *P* < 0.1) and I^2^ statistic (where > 50% indicates significant heterogeneity) [38]. A fixed-effect model was used for comparing the trials without showing heterogeneity, whereas a random effect model was selected for comparing trials showing heterogeneity. Pooled ORs were calculated for allele model, the dominant model, the recessive model and the homozygous model. We performed sensitivity analyses by omitting each study in turn to determine the effect on the test of heterogeneity and evaluated the stability of the overall results. Hardy-Weinberg equilibrium (HWE) was assessed in the controls using Pearson's χ^2^ test. Potential publication bias was investigated with the use of Begger's and Egger's linear regression test [39]; *P* < 0.05 was considered to indicate statistically significant. The power of this meta-analysis for five SNPs was calculated according to the method recommended by Hedges and Pigott [40], with a significant value of 0.05. We calculated the FPRP of this meta-analysis based on the methods of Wacholder et al. [41] and preset an FPRP value of 0.5.

## SUPPLEMENTARY TABLE



## ABBREVIATIONS

IBD: inflammatory bowel disease
CD: Crohn's disease
UC: ulcerative colitis
MYO9B: Myosin IXB
CI: confidence interval
OR: odds ratio
NOS: Newcastle-Ottawa Scale
HWE: Hardy-Weinberg equilibrium
