# Supplementary material for: MYO9B gene polymorphisms are associated with the risk of inflammatory bowel diseases
Source: Oncotarget. 2016 Aug 10;7(37):58862–75. doi: 10.18632/oncotarget.11186 (PMC5312281; doi:10.18632/oncotarget.11186)
Supplement: Supplementary file 1 [file oncotarget-07-58862-s001.docx]

| Author and year | Numbers | |  |  |  |  |  |  |  |  |  |  |  |
| --- | --- | --- | --- | --- | --- | --- | --- | --- | --- | --- | --- | --- | --- |
|  | IBD | |  |  | UC |  |  | CD |  |  | Controls |  |  |
| **rs962917** | GG | | GA | AA | GG | GA | AA | GG | GA | AA | GG | GA | AA |
| Hu_2014 | 38 | | 163 | 241 | 17 | 84 | 134 | 21 | 79 | 107 | 27 | 173 | 207 |
| Wolters_2011 | 176 | | 625 | 556 | 87 | 284 | 232 | 92 | 343 | 319 | 140 | 440 | 344 |
| Latiano_2008 | 442 | | 537 | 215 | 241 | 290 | 124 | 201 | 247 | 91 | 260 | 333 | 81 |
| **rs1457092** | CC | | CA | AA | CC | CA | AA | CC | CA | AA | CC | CA | AA |
| Wolters_2011 | 556 | | 625 | 176 | 232 | 284 | 87 | 319 | 343 | 92 | 344 | 440 | 140 |
| Cooney_2009 | 504 | 612 | | 186 | 273 | 296 | 81 | 252 | 307 | 93 | 533 | 527 | 130 |
| Nunez_2007 | 539 | | 539 | 226 | 258 | 282 | 137 | 281 | 257 | 89 | 403 | 451 | 136 |
| van Bodegraven_2006 | 224 | | 278 | 86 | 111 | 137 | 42 | 113 | 141 | 44 | 729 | 718 | 177 |
| van Bodegraven_2006 | 461 | | 635 | 219 | 192 | 283 | 105 | 269 | 351 | 115 | 944 | 1104 | 323 |
| van Bodegraven_2006 | 289 | | 392 | 133 | 226 | 315 | 109 | 63 | 77 | 24 | 177 | 207 | 61 |
| Amundsen_2006 | 183 | | 212 | 62 | 123 | 143 | 42 | 61 | 69 | 19 | 233 | 258 | 71 |
| **rs1545620** | AA | | AC | CC | AA | AC | CC | AA | AC | CC | AA | AC | CC |
| Hu_2014 | 63 | | 128 | 251 | 37 | 65 | 133 | 26 | 63 | 118 | 42 | 135 | 225 |
| Shi_2011 | 5 | | 94 | 146 | 146 | 94 | 5 | 290 | 355 | 109 | 21 | 104 | 175 |
| Wolters_2011 | 489 | | 651 | 217 | 203 | 294 | 106 | 222 | 317 | 113 | 311 | 450 | 163 |
| Cooney_2009 | 438 | | 634 | 230 | 241 | 310 | 99 | 146 | 251 | 116 | 408 | 578 | 204 |
| Latiano_2008 | 323 | | 568 | 260 | 177 | 317 | 144 | 50 | 144 | 104 | 218 | 348 | 108 |
| van Bodegraven_2006 | 98 | | 284 | 206 | 48 | 140 | 102 | 138 | 361 | 236 | 208 | 747 | 669 |
| van Bodegraven_2006 | 256 | | 648 | 411 | 118 | 287 | 175 | 30 | 80 | 54 | 383 | 1140 | 848 |
| van Bodegraven_2006 | 174 | | 405 | 235 | 144 | 324 | 182 | 26 | 63 | 118 | 77 | 216 | 151 |
| **rs2305764** | GG | | GA | AA | GG | GA | AA | GG | GA | AA | GG | GA | AA |
| Wolters_2011 | 472 | | 657 | 228 | 196 | 296 | 111 | 280 | 359 | 115 | 311 | 450 | 163 |
| Cooney_2009 | 449 | | 631 | 222 | 426 | 200 | 24 | 598 | 53 | 1 | 391 | 582 | 217 |
| Latiano_2008 | 425 | | 541 | 237 | 228 | 301 | 127 | 197 | 240 | 110 | 245 | 339 | 90 |
| Nunez_2007 | 489 | | 569 | 246 | 227 | 300 | 150 | 262 | 269 | 96 | 364 | 469 | 157 |
| van Bodegraven_2006 | 194 | | 287 | 107 | 91 | 143 | 56 | 103 | 144 | 51 | 622 | 766 | 236 |
| van Bodegraven_2006 | 412 | | 648 | 255 | 172 | 288 | 120 | 241 | 360 | 134 | 831 | 1145 | 395 |
| van Bodegraven_2006 | 277 | | 396 | 141 | 215 | 318 | 117 | 62 | 78 | 24 | 165 | 212 | 68 |
| Amundsen_2006 | 154 | | 223 | 80 | 103 | 150 | 55 | 51 | 72 | 26 | 182 | 276 | 104 |
| **rs2305767** | AA | | AG | GG | AA | AG | GG | AA | AG | GG | AA | AG | GG |
| Wolters_2011 | 425 | | 669 | 263 | 203 | 294 | 106 | 228 | 373 | 153 | 322 | 447 | 155 |
| Cooney_2009 | 547 | | 594 | 161 | 266 | 300 | 84 | 249 | 308 | 95 | 407 | 578 | 205 |
| Nunez_2007 | 446 | | 615 | 243 | 263 | 297 | 117 | 183 | 318 | 126 | 296 | 518 | 176 |
| van Bodegraven_2006 | 224 | | 278 | 86 | 118 | 134 | 38 | 107 | 143 | 48 | 507 | 801 | 316 |
| van Bodegraven_2006 | 472 | | 632 | 211 | 221 | 274 | 85 | 251 | 357 | 127 | 814 | 1151 | 406 |
| van Bodegraven_2006 | 297 | | 389 | 128 | 217 | 317 | 116 | 61 | 78 | 25 | 149 | 217 | 79 |
| Amundsen_2006 | 72 | | 219 | 166 | 46 | 146 | 116 | 26 | 73 | 50 | 91 | 271 | 200 |
